# Clinician documentation of patient centered care in the electronic health record

**DOI:** 10.1186/s12911-022-01794-w

**Published:** 2022-03-12

**Authors:** Jorie M. Butler, Bryan Gibson, Olga V. Patterson, Laura J. Damschroder, Corrinne H. Halls, Daniel W. Denhalter, Matthew H. Samore, Haojia Li, Yue Zhang, Scott L. DuVall

**Affiliations:** 1 VA Salt Lake City Health Care System, IDEAS Center of Innovation, George E. Whalen Department of Veterans Affairs Health Care System, 500 Foothill Drive, Salt Lake City, UT 84148 USA; 2 VA Salt Lake City Health Care System, Geriatric Research Education and Clinical Center, George E. Whalen Department of Veterans Affairs Health Care System, 500 Foothill Drive, Salt Lake City, UT 84148 USA; 3 Division of Geriatrics, Department of Internal Medicine, University of Utah, University of Utah School of Medicine, 30 N. 1900 E. AB193 SOM, Salt Lake City, UT 84132-0001 USA; 4 Division of Epidemiology, Department of Internal Medicine, University of Utah, 295 Chipeta Way, Salt Lake City, UT 84132 USA; 5 Department of Biomedical Informatics, University of Utah, 421 Wakara Way, Ste 140, Salt Lake City, UT 84108-3514 USA; 6 Ann Arbor VA Center for Clinical Management Research, P.O. Box 130170, Ann Arbor, MI 48113-0170 USA

**Keywords:** Patient centered care, Medical record review, Contextual information, Goals

## Abstract

**Background:**

In this study we sought to explore the possibility of using patient centered care (PCC) documentation as a measure of the delivery of PCC in a health system.

**Methods:**

We first selected 6 VA medical centers based on their scores for a measure of support for self-management subscale from a national patient satisfaction survey (the Survey for Healthcare Experience-Patients). We accessed clinical notes related to either smoking cessation or weight management consults. We then annotated this dataset of notes for documentation of PCC concepts including: patient goals, provider support for goal progress, social context, shared decision making, mention of caregivers, and use of the patient's voice. We examined the association of documentation of PCC with patients’ perception of support for self-management with regression analyses.

**Results:**

Two health centers had < 50 notes related to either tobacco cessation or weight management consults and were removed from further analysis. The resulting dataset includes 477 notes related to 311 patients total from 4 medical centers. For a majority of patients (201 out of 311; 64.8%) at least one PCC concept was present in their clinical notes. The most common PCC concepts documented were patient goals (patients n = 126; 63% clinical notes n = 302; 63%), patient voice (patients n = 165, 82%; clinical notes n = 323, 68%), social context (patients n = 105, 52%; clinical notes n = 181, 38%), and provider support for goal progress (patients n = 124, 62%; clinical notes n = 191, 40%). Documentation of goals for weight loss notes was greater at health centers with higher satisfaction scores compared to low. No such relationship was found for notes related to tobacco cessation.

**Conclusion:**

Providers document PCC concepts in their clinical notes. In this pilot study we explored the feasibility of using this data as a means to measure the degree to which care in a health center is patient centered. Practice Implications: clinical EHR notes are a rich source of information about PCC that could potentially be used to assess PCC over time and across systems with scalable technologies such as natural language processing.

## Highlights

- Patient centered care integrates patient goals and preferences into clinical care.
- Scalable measures of the delivery of patient centered care by health systems are needed.
- We examined documentation of patient centered care in clinical notes and the association with the health system site score for patient satisfaction with support for self-management.
- Patient centered care concepts were present to varying degrees in clinical notes.
- Natural language processing could be used to identify patient centered care in health systems.

## Background

### Patient centered care

Patient Centered Care (PCC) involves treating the patient as a whole person and engaging the patient in their care [1]. PCC encompasses a broad set of concepts and actvities including (1) activation (involving patients in care, information and goal sharing) (2) relationship building (thoughtful communication, information sharing), and (3) shared decisions (collaborating and incorporating patient preferences at all levels of healthcare delivery) [2, 3]. PCC is a central value in medicine, considered by the National Academy of Medicine to be on par with safety, effectiveness and equity [4, 5]. PCC influences satisfaction with treatment, greater satisfaction with the provider and clinic, less decisional conflict for patients, reduced healthcare costs, and in some cases improved health outcomes [1, 6–9]. Efforts to ensure care is Patient Centered are well underway in a variety of settings. For example, the Veterans Health Administration (VHA) has launched several initiatives to promote PCC including the implementation of Patient Aligned Care Teams and the creation of the Office of Patient Centered Care and Cultural Transformation (OPCC&CT), which promotes care that prioritizes Veteran values [10, 11].

As noted above, PCC is a complex concept and thus challenging to measure, particularly given the multiple levels of healthcare delivery at which it manifests [12, 13]. Current approaches to measure PCC include observation (e.g. either direct observation or via video or audio recording) and coding of patient and provider interactions in primary care clinic encounters. One example is the Roter Interaction Analysis System (RIAS), which is a coding system used to assess PCC that focuses on two-way provider-patient communication including concepts related to building rapport, social talk, and exchanging information [14]. While interaction coding such as the RIAS provides a rich understanding of patient provider interactions, it is a very resource-intensive approach that is not feasible for large-scale use. Another approach used to assess PCC is to survey patients about their perceptions of the patient centeredness of interactions [15, 16]. However, surveys may be burdensome for patients, and are subject to the biases to which all self reported data are prone (e.g. selection bias, desirability bias, recall bias, etc.). To complement current methods of measuring PCC, additional measurement methods are needed. To date very little research has examined the use of clinical documentation as a means to measure the delivery of PCC.

Clinical documentation may reflect an important information source for assessing the extent to which PCC occurs in clinical practice. Clinical notes may be an important indicator of provider conceptions of the patient including patient activation, patient goals and preferences, patient "voice" (quotes from patients in notes), and informational priorities. However, because this information is not currently recorded as structured data within the electronic health record (EHR), it can be challenging to measure the extent to which PCC occurs in clinical practice [3].

Measuring the degree to which PCC concepts are available in clinical documentation is the first step toward determining if natural language processing of clinical notes could provide a scalable method to measure PCC. In the current study, we sought to measure the documentation of PCC in the EHR within VHA. We also sought to evaluate whether the documentation of PCC was associated with patient satisfaction at the health center.

### Study objectives

Our objective in this study was to first examine the degree to which PCC concepts were documented in clinical notes and then to explore the association between PCC documentation in clinical notes and patient satisfaction with behavioral health support at the health center. We hypothesized that greater documentation of PCC would be associated with a higher SHEP subscale score.

## Methods

All procedures in this study were approved by the University of Utah Institutional Review Board and the Salt Lake City VA Medical Center Research Review Board. A waiver of HIPAA Authorization for Research was approved.

### Annotation guide

PCC requires consideration of the patient as a “whole person”. Measurement of this construct requires operationalizing several features. These include attention to contextual information about the patient’s life, including social support and cultural and spiritual beliefs, which are important for adapting treatment plans to meet the needs of patients [17, 18]. Another key facet of PCC is assessing patient goals to understand the patient’s perspective and desires. PCC communication may be captured when the provider uses quotes or other explicit references to the patient’s comments in the EHR. This type of documentation, referred to as patient voice, may be an indication that the provider was listening to the patient [19].

For this study we identified seven target PCC concepts from the literature [18, 20, 21]: intentions or goal setting, evidence of progress towards goals, presence of caregiver, provider support towards goals, shared decision-making, social context, and patient voice (Table 1). Our research team developed guidelines for defining concepts based on work in prior literature and the general approach that PCC involves assessing patient goals, using shared decision making, and assessing contextual variables. See “Appendix” for a detailed description of the concepts and their attributes. Four experienced clinical abstractors were trained to identify evidence of PCC. Abstractors included an informaticist, a health psychologist, and two full-time chart abstractors with clinical research backgrounds. The informaticist and health psychologist first annotated the clinical notes in the study. An attempt was then made to calibrate with the other half (clinical research background) of the abstractor team, but interrater agreement was low (< 20% agreement) and given our feasibility pilot status we chose to focus instead on a consensus approach, coding separately and resolving any disagreement by discussion.

**Table 1:** (A) Patient charts with at least one PCC Concept. (B) Notes with at least one PCC Concept

	Weight Loss (N = 100)	Smoking Cessation (N = 101)	Total (N = 201)
(A)			
Goal intention	84 (84%)	42 (41.6%)	126 (62.7%)
Mention of caregiver	5 (5%)	0 (0%)	5 (2.5%)
Progress toward tobacco cessation	1 (1%)	44 (43.6%)	45 (22.4%)
Progress toward weight loss	63 (63%)	12 (11.9%)	75 (37.3%)
Provider support for goal progress	52 (52%)	72 (71.3%)	124 (61.7%)
Shared decision making	6 (6%)	29 (28.7%)	35 (17.4%)
Social context	57 (57%)	48 (47.5%)	105 (52.2%)
voice	74 (74%)	91 (90.1%)	165 (82.1%)

	Weight Loss (N = 322)	Smoking Cessation (N = 155)	Total (N = 477)
(B)			
Goal intention	240 (74.5%)	62 (40%)	302 (63.3%)
Mention of caregiver	7 (2.2%)	0 (0%)	7 (1.5%)
Progress toward tobacco cessation	1 (0.3%)	56 (36.1%)	57 (11.9%)
Progress toward weight loss	192 (59.6%)	22 (14.2%)	214 (44.9%)
Provider support for goal progress	94 (29.2%)	97 (62.6%)	191 (40%)
Shared decision making	7 (2.2%)	30 (19.4%)	37 (7.8%)
Social context	120 (37.3%)	61 (39.4%)	181 (37.9%)
Voice	200 (62.1%)	123 (79.4%)	323 (67.7%)

### Cohort selection

VHA is one of the world’s largest integrated health care systems with over 170 stations across the United States and US territories. To qualify for VHA care, patients must have served in the US military and must apply for the benefit of healthcare. VHA medical centers provide a broad spectrum of healthcare including primary care, critical care, radiology, surgery, and mental health services to over 9 million US Veterans annually [22]. In an effort to provide a central repository for clinical research and quality analysis, data from all VHA sites are aggregated into the Corporate Data Warehouse (CDW). The CDW mission is to provide access to data by standardizing, consolidating, and streamlining clinical data systems for improving quality of care and research. The CDW is made available to VA-affiliated researchers through VA Informatics and Computing Infrastructure (VINCI). These data include coded and administrative data along with clinical notes created by providers and adminstrators in the course of patient care. VINCI provides secure access to diverse types of data including access to Textual Integration Utilities (TIU) notes, which are clinical notes from free text stored in the EHR (e.g., progress notes). Data access for research is obtained through a permissions process requiring ethics approval and review. Data analyses tools are available for use within the VINCI workspace [23].

To support investigation of a relationship between documentation of PCC concepts in the EHR and patient satisfaction, we used data from the Survey for Healthcare Experiences of Patients (SHEP) [24]. Managed by the VHA Office of Quality and Performance, SHEP is regularly offered and is used to assess patients’ responses at their most recent episode of care. Studies have used SHEP data to assess relationships between facets of care (e.g., primary care provider turnover) and patient satisfaction [25]. For this study, we used scores from the self management support section of the SHEP, which asks about the perceptions the patient has about being involved in their care. We used this subscore because we planned to access clinical notes that were related to weight management (MOVE) and tobacco cessation; health issues requiring behavior change and self-management (areas where we hypothesized that documentation of PCC constructs might be enriched). We believed that the self management support subscore of the SHEP would be more reflective of "patient satisfaction" in this particular domain, expected to relate most closely to health issues like weight and tobacco. We selected three VA centers with high SHEP self management support scores and three with low scores.

We analyzed records for patients who sought clinical care for weight loss and smoking cessation as indicated by a consultation request from a primary care provider for these services over a one-year period. These service types were chosen as a reasonable target for an initial test of PCC documentation given that they involve health issues related to behvaioral change and require self-management.

**Fig. 1:**
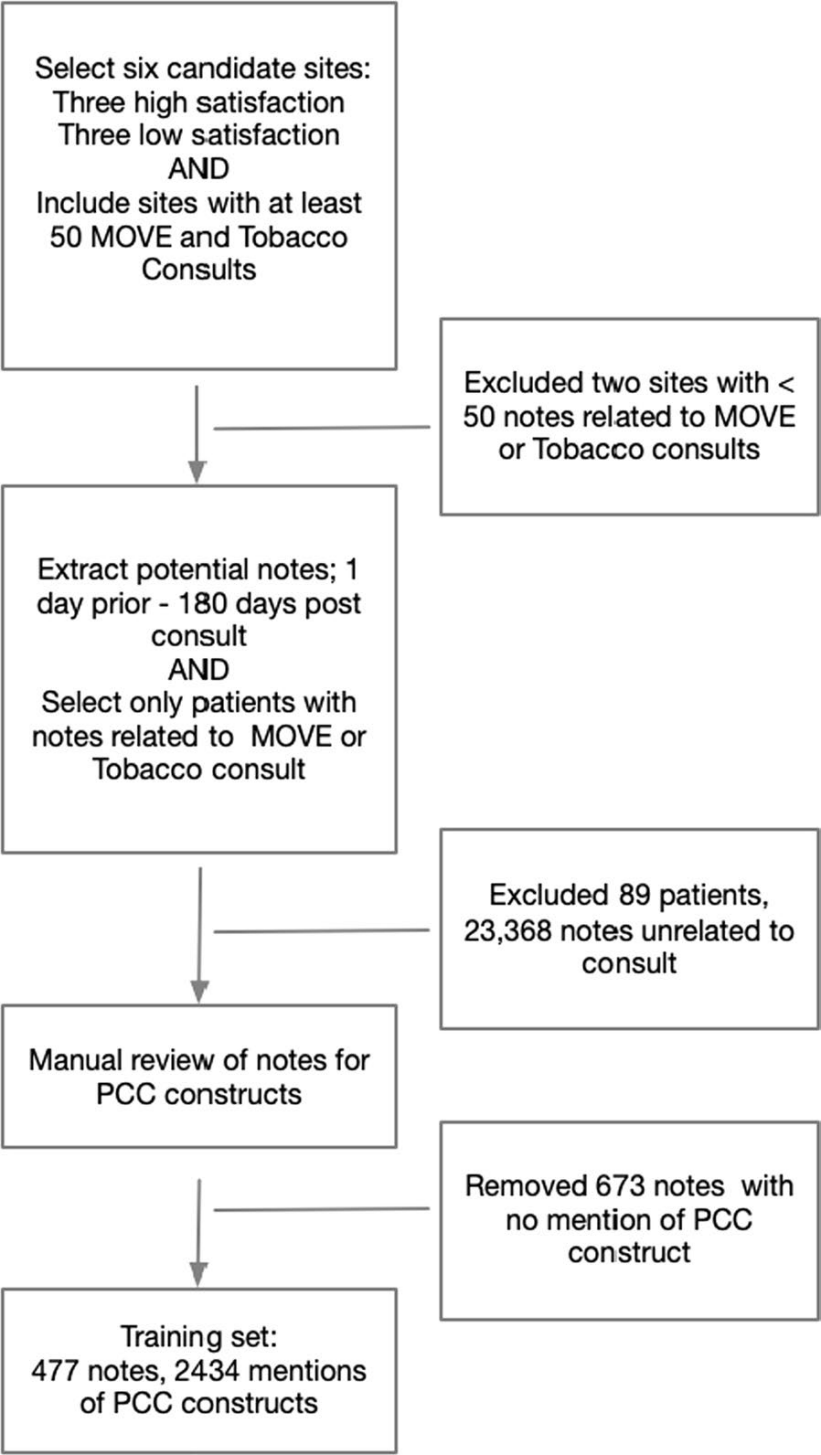
Note Selection Process

We excluded from analysis data from sites that had < 50 completed consultations in either the weight loss or tobacco cessation category (see Fig. 1). From the remaining sites, we randomly selected 50 patients with weight loss and 50 patients with tobacco cessation consultation requests. For these patients, all clinical notes in the EHR created the day before and up to 180 days after the consultation visit were identified (24,518 notes in total). We chose this 180 day interval to keep the number of notes manageable for a pilot study. Then, we selected 58 note titles that would indicate a note relevant to the weight loss or tobacco cessation consult (e.g., weight, nutrition, smoke, tobacco cesssation). Review and abstraction were performed using the Extensible Human Oracle Suite of Tools (eHost) [26], developed by a research team in VA. This tool was accessed and used with the VINCI workspace [27]. The eHost tool is open source and designed to organize annotations to clinical notes documents. The tool allows for the organization of projects and files and permits the export of annotation data to excel for analyses. The tool is available with additional information and open access on GitHubb [28]. Each mention of any of the identified PCC concepts was reviewed and labeled with the appropriate classes and attributes.

## Results

Of the 311 patients with relevantly titled notes, 201 (64.6%) had at least one clinical note that documented a PCC concept. There were a total of 2434 PCC concepts identified across 477 weight loss or tobacco cessation clinical notes for these patients. *Patient voice* (specific quotes or references to patient remarks, suggesting listening to the patient) was the most common concept documented within the clinical notes (see Table 1). Other frequently documented concepts included *references to patient goals, provider support for goal progress*, and *social context*. *Shared decision-making* and *mention of a caregiver* were seldom documented in the EHR. Density of PCC concepts varied by type of clinical note. The *goal intention* concept appeared at least once in the weight loss clinical notes for 84 patients (84%); and at least once in the smoking cessation clinical notes for 42 patients (42%). *Specific progress toward a goal* (weight loss or smoking cessation) was concordant with note type. *Provider support for goal progress* was documented among 72 patients (71%) with smoking cessation clinical notes and 52 patients (52%) with weight loss clinical notes. *Patient voice* and *shared decision making* were found among a higher proportion of patients with smoking cessation clinical notes ((91 patients (90%) and 29 patients (28%) respectively)) compared to patients with weight loss clinical notes ((74 patients (74%) and 6 patients (6%)). Examining the number of instances annotated per concept demonstrated a relatively large range for some concepts such as *goal intention, social context* and *patient voice* (See Table 1(A)). We found that for almost all concepts (except social context and caregiver) there were more annotations related to weight loss notes compared to smoking cessation notes.

Examining the pattern of annotation of PCC concepts across sites revealed that across the three high scoring SHEP sites included in the analysis, there were an average of 53 patients out of 100 per station with qualifying notes (note titles relating to the specific consult visit), with a total of 365 PCC concepts documented, and an average of 6.4 PCC concepts documented per patient (see Table 1(A)). At the low SHEP scoring site with a sufficient number of qualifying notes, 41 patients out of 100 randomly selected had qualifying notes, with a total of 215 PCC concepts documented, and an average of 5.2 PCC concepts documented per patient. When we compared results statistically using poisson regression analyses all 3 high satisfaction sites had significantly more clinical notes per patient documenting patient goals in weight loss notes (see Table 2(B)) compared to the lower satisfaction site. We also found one higher satisfaction site had significantly more clinical notes per patient documenting progress toward weight loss and documenting patient voice in weight loss related notes compared to the lower patient satisfaction site. This particular high satisfaction site also demonstrated an overall effect of a higher number PCC concepts documented per patient per weight loss note compared to the lower satisfaction site. We found no relationships in the tobacco related notes.

**Table 2:** (A) Number of EHR notes with PCC concepts per patient by satisfaction with at least one PCC concept. (B) Number of EHR notes with PCC concepts per patient by site with at least one PCC concept

PCC concept	M-L	M-H	T-L	T-H
(A)				
Goal intention				
Mean (SD)	3 (4.3)	2.8 (3.1)	1 (0)	1.6 (1.4)
Median (IQR)	1 (1.5)	1 (2)	1 (0)	1 (0)
Range	(1, 15)	(1, 18)	(1, 1)	(1, 6)
*P* value		0.739		0.222
Mention of caregiver				
Mean (SD)	–	1.4 (0.5)	–	–
Median (IQR)	–	1 (1)	–	–
Range	–	(1, 2)	–	–
*P* value		–		–
Progress Toward Tobacco Cessation				
Mean (SD)	1 (NA)	–	1.2 (0.4)	1.3 (0.7)
Median (IQR)	1 (0)	–	1 (0)	1 (0)
Range	(1, 1)	–	(1, 2)	(1, 4)
*P* value		–		0.651
Progress Toward Weight Loss				
Mean (SD)	2.7 (3.7)	3.1 (2.9)	1 (NA)	1.9 (1.6)
Median (IQR)	1 (1)	2 (3)	1 (0)	1 (1.5)
Range	(1, 13)	(1, 16)	(1, 1)	(1, 5)
*P* value		0.742		0.531
Provider Support For Goal Progress				
Mean (SD)	1.1 (0.4)	1.9 (1.2)	1.3 (0.6)	1.4 (1.1)
Median (IQR)	1 (0)	1 (2)	1 (0.2)	1 (0)
Range	(1, 2)	(1, 5)	(1, 3)	(1, 8)
*P* value		0.164		0.892
Shared Decision Making				
Mean (SD)	–	1.2 (0.4)	1 (0)	1 (0.2)
Median (IQR)	–	1 (0)	1 (0)	1 (0)
Range	–	(1, 2)	(1, 1)	(1, 2)
*P* value		–		0.942
Social Context				
Mean (SD)	2.4 (4.3)	2 (1.4)	1.1 (0.3)	1.3 (1)
Median (IQR)	1 (0)	1.5 (2)	1 (0)	1 (0)
Range	(1, 14)	(1, 6)	(1, 2)	(1, 5)
*P* value		0.488		0.469
Voice				
Mean (SD)	2.5 (3.7)	2.7 (3)	1.3 (0.7)	1.4 (0.9)
Median (IQR)	1 (1)	1 (2)	1 (0)	1 (0)
Range	(1, 14)	(1, 18)	(1, 3)	(1, 7)
*P* value		> 0.99		0.822
All Concepts				
Mean (SD)	2.8 (3.7)	3.3 (3.6)	1.4 (0.7)	1.6 (1.3)
Median (IQR)	1 (1)	2 (3)	1 (0.5)	1 (1)
Range	(1, 15)	(1, 21)	(1, 3)	(1, 9)
*P* value		0.859		0.528

PCC Concept	M-L	M-H1	M-H2	M-H3	T-L	T-H1	T-H2	T-H3
(B)								
Goal Intention								
Mean (SD)	3 (4.3)	1.8 (1)	1.8 (1.6)	4.7 (4.4)	1 (0)	1.5 (1.3)	1.2 (0.4)	1.9 (1.8)
Median (IQR)	1 (1.5)	1.5 (1.2)	1 (1)	3 (7)	1 (0)	1 (0)	1 (0)	1 (0.5)
Range	(1, 15)	(1, 4)	(1, 7)	(1, 18)	(1, 1)	(1, 6)	(1, 2)	(1, 6)
*P* value		0.048*	0.025*	0.025*		0.293	0.766	0.12
Mention Of Caregiver								
Mean (SD)	–	1.3 (0.6)	–	1.5 (0.7)	–	–	–	–
Median (IQR)	–	1 (0.5)	–	1.5 (0.5)	–	–	–	–
Range	–	(1, 2)	–	(1, 2)	–	–	–	–
*P* value		–	–	–		–	–	–
Progress Toward Tobacco Cessation								
Mean (SD)	1 (NA)	–	–	–	1.2 (0.4)	1.4 (0.5)	1.3 (0.6)	1.3 (0.8)
Median (IQR)	1 (0)	–	–	–	1 (0)	1 (1)	1 (0)	1 (0)
Range	(1, 1)	–	–	–	(1, 2)	(1, 2)	(1, 3)	(1, 4)
*P* value		–	–	–		0.673	0.792	0.672
Progress Toward Weight Loss								
Mean (SD)	2.7 (3.7)	2.5 (1.3)	2.2 (1.8)	4.3 (3.9)	1 (NA)	1.5 (1.2)	1 (0)	3.3 (2.1)
Median (IQR)	1 (1)	2.5 (3)	1.5 (1.8)	3 (5)	1 (0)	1 (0)	1 (0)	4 (2)
Range	(1, 13)	(1, 4)	(1, 7)	(1, 16)	(1, 1)	(1, 4)	(1, 1)	(1, 5)
*P* value		0.8	0.442	0.021*		0.7	> 0.99	0.251
Provider Support For Goal Progress								
Mean (SD)	1.1 (0.4)	2.4 (1.4)	1.6 (1)	1.9 (1.2)	1.3 (0.6)	1.4 (0.9)	1.2 (0.4)	1.4 (1.7)
Median (IQR)	1 (0)	2 (2.8)	1 (0.5)	1 (1)	1 (0.2)	1 (0)	1 (0)	1 (0)
Range	(1, 2)	(1, 5)	(1, 4)	(1, 5)	(1, 3)	(1, 5)	(1, 2)	(1, 8)
*P* value		0.066	0.441	0.221		0.875	0.766	0.744
Shared Decision Making								
Mean (SD)	–	1 (NA)	1 (NA)	1.2 (0.5)	1 (0)	1.1 (0.3)	1 (0)	1 (0)
Median (IQR)	–	1 (0)	1 (0)	1 (0.2)	1 (0)	1 (0)	1 (0)	1 (0)
Range	–	(1, 1)	(1, 1)	(1, 2)	(1, 1)	(1, 2)	(1, 1)	(1, 1)
*P* value		–	–	–		0.87	> 0.99	> 0.99
Social Context								
Mean (SD)	2.4 (4.3)	1.6 (1)	1.8 (1.4)	2.7 (1.5)	1.1 (0.3)	1.2 (0.9)	1.5 (0.7)	1.5 (1.2)
Median (IQR)	1 (0)	1 (1)	1 (1)	2.5 (1.5)	1 (0)	1 (0)	1.5 (0.5)	1 (0)
Range	(1, 14)	(1, 4)	(1, 6)	(1, 6)	(1, 2)	(1, 5)	(1, 2)	(1, 5)
*P* value		0.183	0.25	0.718		0.655	0.602	0.386
Voice								
Mean (SD)	2.5 (3.7)	1.7 (1)	2 (1.9)	4.3 (4.1)	1.3 (0.7)	1.3 (0.9)	1.6 (0.6)	1.3 (1.2)
Median (IQR)	1 (1)	1 (1)	1 (1)	2 (5.5)	1 (0)	1 (0)	1.5 (1)	1 (0)
Range	(1, 14)	(1, 4)	(1, 8)	(1, 18)	(1, 3)	(1, 5)	(1, 3)	(1, 7)
*P* value		0.142	0.301	0.011*		0.966	0.511	0.922
All Concepts								
Mean (SD)	2.8 (3.7)	2.4 (1.3)	2 (1.8)	5.4 (4.9)	1.4 (0.7)	1.5 (1)	1.8 (0.9)	1.6 (1.7)
Median (IQR)	1 (1)	2 (2.2)	1 (1)	3 (8)	1 (0.5)	1 (0.8)	1.5 (1.2)	1 (0)
Range	(1, 15)	(1, 5)	(1, 9)	(1, 21)	(1, 3)	(1, 6)	(1, 3)	(1, 9)
*P* value		0.41	0.093	< 0.001*		0.82	0.348	0.6

In (A) and (B) the column names stand for the combination of Weight Loss (M) / Smoking Cessation (T) and Satisfaction (L for Low and H for Hight) or Site (L for the only one with low satisfaction and H1-H3 for the other three with high satisfaction)

In both tables, the low satisfaction site is considered as the reference level. We compare L with all the three high satisfaction sites together as the high satisfaction group in (A) and compare L with each of the three high satisfaction sites separately in (B). The *P* values are produced using Poisson regression. In (A), the random effect account for the clustering effect among patients from the same site is also added to the regression model

(A) Shows that there is no significant difference between satisfaction levels, while in (B) we do detect some significant differences under the Weight Loss group, including L vs H1, L vs H2 and L vs H3 for Goal Intention, and L vs H3 for Progress Toward Weight Loss, Voice and All Concepts.

## Discussion

In this project we examined the documentation of PCC concepts in notes where we might expect these concepts to be more likely to be documented (weight loss and tobacco cessation related clinical notes focused on health issues that require behavioral change and/or self management). We also explored the association between the prevalance of PCC documentation and the health center’s score on a measure of support for self management in a national survey of patient satisfaction. We found that the majority of patients had notes with PCC concepts documented and the documentation of PCC concepts was higher in sites with higher scores on support for self management. This work demonstrates that PCC concepts can be systematically identified within clinical notes addressing health issues which require behavioral change or greater self-management. Some concepts with high density such as patient goals, social context, and patient voice (indicating documentation of direct patient comments suggesting close listening) may be central to providers’ mental representations of the patient's care and therefore be documented.

Our study design presumes that providers who are more patient centered reflect that patient centeredness in their documentation. Other work has demonstrated links between clinical reasoning, clinician goals, and EHR documentation [29]. Our presumption may be reasonable given that providers who consider PCC to be important may want reminders of where they left off with the patient for the next encounter (e.g., to remind them of a previously discussed patient goal). However, further research is needed to confirm a relationship between PCC visit behavior and PCC documentation in clinical notes.

Our statistical analyses showed that for weight loss notes the documentation of patient weight loss goals was higher at all 3 sites with high satisfaction with self-management scores compared to the low satisfaction site. In addition, at one higher satisfaction site there were more clinical notes per patient documenting patient voice, progress toward weight loss, and overall PCC concepts. This suggests that sites with on average higher satisfaction scores for support for patient self-management have greater PCC documentation for weight loss notes. This result is consistent with models of PCC pointing to the centrality of patient goals in the process [30–32].

We did not find similar relationships for tobacco cessation notes. Patients who smoke may potentially be different in distinct ways that lead providers to record documentation in EHR notes differently. These patients may be more ill [33] or may even be more apt to have personality characteristics that may influence their interactions with providers [34]. Other research has found associations between tobacco cessation discussions in primary care and higher satisfaction with healthcare [35]. Potentially the more homogenous documentation density in tobacco cessation is the key factor, but it is not clear why this would be the case.

### Patient centered care and informatics

Informatics is well suited to address some of the complex problems that must be addressed if healthcare is going to be more patient centered. These include enhancing patient engagement, facilitating information exchange across all stakeholders in the complex healthcare system, and facilitating shared decision making [36, 37]. Informatics solutions proposed to enhance patient centered care include interventions to support patient activation via social media [38], developing informatics systems to support coordination of care [39, 40], supporting patient access to health data including access to clinical notes through OpenNotes [41, 42], and systems [43] intended to help with shared decision making [44, 45]. The US goal for meaningful use within the electronic health record (EHR) mandates greater attention to PCC in EHR design [46].

This pilot study demonstrates that reviewing EHR notes may be an important addition to measurement techniques in examining PCC, and has future potential as a useful tool for demonstrating the value of PCC. Our pilot results suggest that in the future we may be able to use methods like natural language processing to examine PCC at a scale impossible with current measures. This approach has been used in other areas of complexity including identifying information from family member history in the EHR [27]. Natural language processing has also been used to identify social determinants of health in the EHR [47, 48]. Thus, with further developmental work we expect this to be possible.

In addition, this study has implications related to EHR design. One of the most challenging aspects in retrospective investigations on various aspects of patient care is the inability to enforce relevant data entry in a consistent format. Data collection relies on manual review and abstraction of clinical data entered as a byproduct of routine patient care. We know that EHRs currently are not designed to document patient goals or other PCC information as structured data [3]. Future EHR design should address this gap by incorporating structured documentation of patient goals, patient voice and patient preferences; making this information easily searchable might both facilitate the delivery of PCC by providers and the measurement of PCC by health systems. If this change were made future research related to PCC and documentation could focus on harder-to-capture aspects of PCC such as patient contextual data [49]. The EHR can be an impediment to provision of and tracking of PCC. These weaknesses should be addressed in the next generation of EHR design.

### Limitations

Our small, pilot study has several limitations. The self-management support satisfaction scores are measures of patient perceptions of visits within the health system and not necessarily of the specific providers or patients for whom documentation of PCC concepts in clinical notes were assessed. That we found statistical associations between the low satisfaction site and high satisfaction sites suggests this is an area that may benefit from future exploration with more direct linkages between satisfaction measures for specific providers and visits and documentation for those visits in future work. Another limitation is that we do not know whether our findings between higher PCC documentation levels and higher SHEP scores are valid across all patient groups, including groups that are historically underrepresented and/or marginalized within healthcare delivery. This will be an important future direction to pursue. Another limitation is that we used consensus coding rather than establishing rigorous interrater reliability between annotators. In order to scale up this work it will be necessary to refine our initial annotation guide until it is possible to achieve strong interrater reliability for all measured concepts. A final limitation is that we did not have permission to access demographic information about the patients involved which limited the analyses we could perform.

In this pilot work the notes we collected came from a relatively brief time window (181 day period). Future work should consider examining longitudinal relationships and assessing patient provider dyads over time, and the documentation styles of specific providers, which could have implications for scaling up this work. Finally, the types of clinical notes used in this study represent a narrow window of clinical notes. This may have influenced the findings, such as the infrequent documentation of shared decision-making or caregiver mentions. Patients seeking a visit for health issues related to behavioral change may already have engaged in shared decision-making prior to the referral. It will be important to explore how PCC concepts are documented with a broader selection of clinical note types to understand the full utility of PCC documentation as a complement to observational coding and patient surveys.

## Conclusions

Providers document PCC concepts in their clinical notes and there are associations between this documentation and patient satisfaction at a site level. There is potential to use manual chart review to provide effective feedback to clinicians and administrators to make organization-wide policy changes and to potentially improve patient care processes and to examine the impact on patient health outcomes.

To incorporate PCC in care it is essential for healthcare systems and providers to assess the impact of PCC on healthcare outcomes. The approach used here is an early effort to measure PCC in clinical practice.

## Data Availability

In accordance with VA policy and the VA Information Security Officer (ISO) requirements only study team personnel explicitly authorized by data stewards will have access to the data. Those interested in the requisite approvals should contact the corresponding author at jorie.butler@hsc.utah.gov.

## Appendix

Manual chart review framework:

**Table:**

Concept	Concept description or subtype	Subtype description or options	Example
Patient goal or intention is a patient’s statement on his or her goals related to weight loss or smoking cessation	Type	Behavioral goal related to the process of achieving it	“I want to watch my diet”
		Outcome oriented related to an end result	“I want to lose 10 pounds”
	Voice, in which the goal was documented	Provider	Encouraged patient to … “Recommended patient …"
		Discussion	"Discussed", "Reviewed", "Talked about", "Conferred", "Chatted"
		Patient (stated by patient or by provider)	“I want to lose 10 pounds” "Patient says/reports he wants to quit smoking."
	Specificity of goals	Any specific duration or intencity of the goal were stated	“I want to lose 10 pounds” “I want to reduce my calories to 1700 a day”
	Temporality of goals	Mentions of specific frequency related to the stated goal	“Patient plans to eat 3 meals each day"
	Efficacy of the goals	Expressions of the patient’s confidence that they can perform the behavior	"I feel positive I can do X"
Progress toward a goal refers to statement of patient’s specific steps toward achieving a goal	Type	Behavioral change that supports the goal	“Started using nicotine patch”
		Specific outcomes	“Down to one smoke a day” “Lost 5 pounds last month”
	Voice, in which the goal was documented	Provider, discussion, or patient	
Caregiver mention	Any mention of a caregiver or someone who takes on that role or who comes with patient to appointments		"My daughter helps me” "Patient's wife is also his caregiver"
Provider support for goal	Referral	Referral to another specialist that is meant to help patient in achieving goals or motivating	“Referred to cardiologist”
	Other	Other statements referring to provider support	"Praised patient on his recent weight loss", "Reviewed goals with patient"
Shared decision-making	Discussion	Mention of more than one treatment options discussed between patient and provider	"After discussion with patient, patient prefers xyz"
Social context	A broad description of a patient’s social environment related to several categories	Access to care	“Hard to get to appointments”; “Neighbor or family member available for transportation to doctor appointments”; “Lives too far away”; “Barriers to self care”; “Can't get time off from work”
		Social support/marital status	“Lives with family member”; “Married for x number of years”; “Patient recieves help or social support via church, neighborhood, or other organizations”; “Participates in social activities”
		Competing responsibilities	“Caregiver for chronically ill spouse”; “Works full time”; “Cares for several pets”; “Has disabled child living at home”; “Has gone back to school”; “Homeless person needing to focus on survival”
		Relationship with health care providers or provider offers description of patient's social attributes, personality, etc	“Doesn't like a doctor”; “Doesn't feel listened to by a doctor”; “Appreciates a particular provider”; “Feels listened to”; "Patient is pleasant and outgoing"; "Patient was combative"
		Skills and abilities, such as details about past employment or training or mention of education or special skillset	"Patient worked as a mechanical engineer until retirement 10 years ago"
		Emotional state that is expressed as any descriptors of emotional state that are not a part of a standardized questionaire the clinicians use to screen for mental health problems or a mention of acute or chronic mood states	Anxious, worried, nervous, positive, happy
		Financial situation, including employment information, such as disability inquiries; housing status; financial status in general; homelessness; current job	“Patient recently lost his job and is currently receiving food stamp assistance"
		Cultural beliefs and attitudes	Use of complementary or alternative supplements and/or therapies, political views, and practice of holiday traditions
		Spiritual beliefs	Religious upbringing and religious or spirutal views related to a higher power, prayer, afterlife, and marriage or divorce
		Attitude toward illness, but not other life circumstances	Feeling distressed, worried, anxious, positive, hopeful
		Other social context	Such as hobbies, activities, or anything else that relates to social context but is not represented in the other categories
Voice stated without goals	A mention of a patient’s or provider’s statement that is not related to a specific goal	Patient statement	“Veteran stated he has slept more this week”, “I can’t get a job”
		Conversation or discussion statement	“Reviewed assisted living options”; “Talked about patient’s concern about unemployment benefits running out”

## Abbreviations

PCC: Patient centered care
VA: Veterans Affairs
VHA: Veterans Health Administration
OPCC&CT: Office of Patient Centered Care and Cultural Transformation
EHR: Electronic Health Record
CDW: Central Data Warehouse
VINCI: VA Informatics and Computing Infrastructure
SHEP: Survey for Healthcare Experiences of Patients
IRB: Institutional Review Board
US: United States
HIPAA: Health Insurance Portability and Accountability Act
